# Treatment of penetrating trauma of the extremities: ten years’ experience at a dutch level 1 trauma center

**DOI:** 10.1186/1757-7241-21-2

**Published:** 2013-01-14

**Authors:** Oscar JF Van Waes, Esther MM Van Lieshout, Wouter Hogendoorn, Jens A Halm, Jefrey Vermeulen

**Affiliations:** 1 Department of Surgery-Traumatology, Erasmus MC, University Medical Center Rotterdam, P.O. Box 2040, Rotterdam, the Netherlands

## Abstract

**Background:**

A selective non-operative management (SNOM) has found to be an adequate and safe strategy to assess and treat patients suffering from penetrating trauma of the extremities (PTE). With this SNOM comes a strategy in which adjuvant investigations or interventions are not routinely performed, but based on physical examination only.

**Methods:**

All subsequent patients presented with PTE at a Dutch level I trauma center from October 2000 to June 2011 were included in this study. In-hospital and long-term outcome was analysed in the light of assessment of these patients according to the SNOM protocol.

**Results:**

A total of 668 patients (88.2% male; 33.8% gunshot wounds) with PTE presented at the Emergency Department of a level 1 traumacenter, of whom 156 were admitted for surgical treatment or observation. Overall, 22 (14%) patients that were admitted underwent exploration of the extremity for vascular injury. After conservative observation, two (1.5%) patients needed an intervention to treat (late onset) vascular complications. Other long-term extremity related complications were loss of function or other deformity (n = 9) due to missed nerve injury, including 2 patients with peroneal nerve injury caused by delayed compartment syndrome treatment.

**Conclusion:**

A SNOM protocol for initial assessment and treatment of PTE is feasible and safe. Clinical examination of the injured extremity is a reliable diagnostic 'tool' for excluding vascular injury. Repeated assessments for nerve injuries are important as these are the ones that are frequently missed and result in long-term disability. Level of evidence: II / III, retrospective prognostic observational cohort study Key words Penetrating trauma, extremity, vascular injury, complications.

## Background

Penetrating trauma of the extremities (PTE) is considered a difficult injury to manage because artery and nerve injuries can be serious and may significantly impair outcome of the patient. PTE accounts for about 50% of penetrating trauma. Despite possible (long-term) complications, overall survival is very high [1,2]. Nevertheless, the low incidence of this kind of trauma in Western Europe makes it difficult for trauma surgeons to gain experience in its management.

A selective non-operative management (SNOM) has found to be an adequate and safe strategy to assess and treat patients suffering from PTE [3-6]. With this SNOM comes a strategy in which diagnostic computed tomography angiography (CTA) screening is not routinely performed, but based on physical examination only. The accuracy of physical examination to detect vascular injury is very high in patients after penetrating trauma [3,7]. Hard signs of a vascular injury (Table 1) mandate emergent surgical exploration, or, if the patient is hemodynamically stable, endovascular treatment could be considered. Diagnostic CTA is indicated in hemodynamically stable patients with clinical signs of vascular injury (Table 1). Without signs of vascular impairment in PTE a conservative observational strategy without CTA is viable [5,6,8].

**Table 1:** **Signs of arterial injury**[3]

**Hard signs**	Active hemorrhage
	Absent distal pulses or ischemia
	Expanding or pulsatile hematoma
	Bruit or thrill
**Subtle signs**	Subjective reduced or unequal pulses
	Large non-pulsatile hematoma
	Orthopedic injuries carrying a high index of suspicion of vascular injury
	Neural injury
	History of large hemorrhage on trauma scene

The present study was undertaken to assess SNOM in relation to long-term outcome and complications.

### Patients and methods

All patients presented with PTE at a single Dutch level I trauma center from October 2000 to June 2011 were included in this study. Data regarding age, gender, mechanism of injury, type of injury (i.e. vascular, orthopaedic, or nerve), anatomical location and concomitant injuries, clinical manifestations and vital parameters, indications for additional investigations, and treatment strategy of all patients were collected and analyzed in the light of patient’s long-term outcome.

Appropriate approval of the local ethical review board was obtained prior to inclusion of patients into the trauma database. Due to the retrospective and anonymous nature of the database no further approval was deemed necessary by the ethical review board.

**Figure 1:**
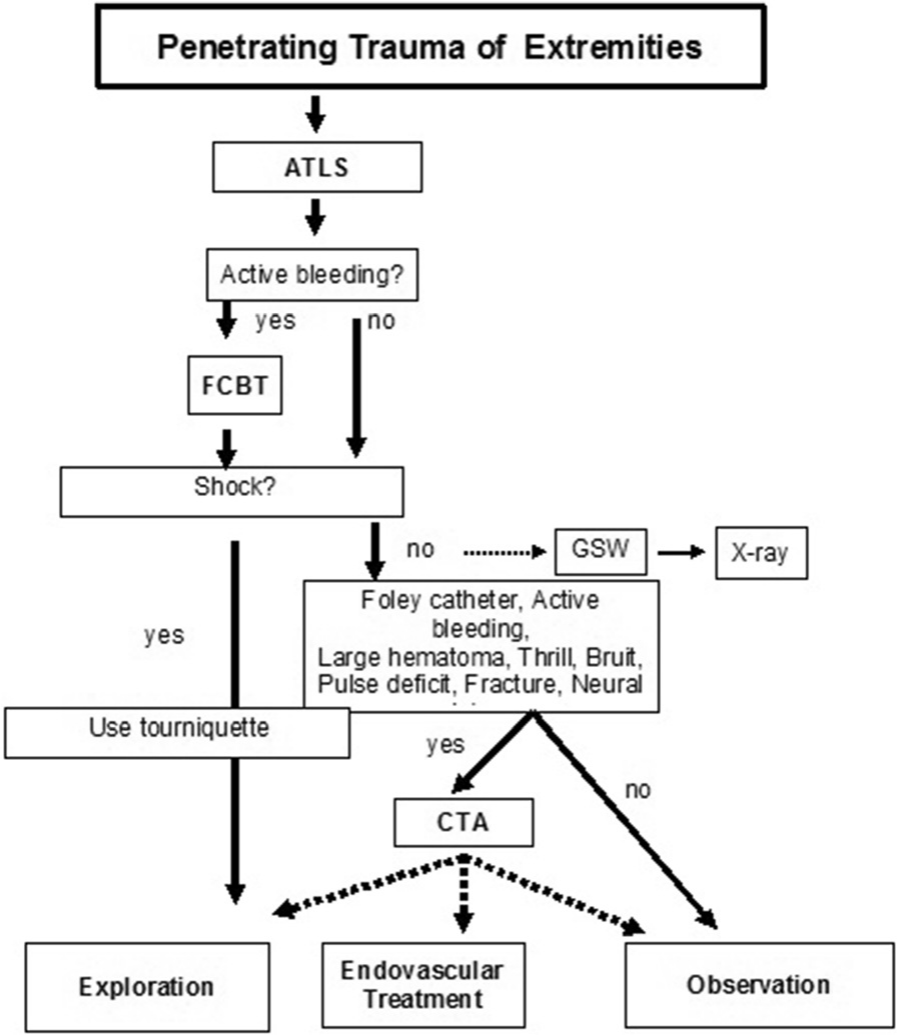
**Algorithm for initial management of patients with penetrating trauma of extremities.** ATLS = Advanced Trauma Life Support; CTA = Computed tomography angiography, FCBT = Foley Catheter Balloon Tamponade, GSW = Gun Shot Wound, CTA = Computed Tomographic Angiography.

All patients were initially resuscitated according to the Advanced Trauma Life Support (ATLS®) [9] guidelines and to the discretion of the trauma surgeon in charge. A local protocol was established in order to manage these injuries (Figure 1): Hemodynamically stable patients, and patients who stabilize after immediate simple resuscitation, were first evaluated with a thorough history and physical examination. Additional diagnostic investigations were performed when indicated by the preset protocol based on history and clinical manifestations. A routine X-ray of the injured extremity was made in patients with a gunshot wound (GSW). Indication for CTA was based on the presence of signs and symptoms of vascular injury found by clinical examination. Patients were immediately transferred to the operating room for surgical intervention if additional severe injuries in need of immediate surgical were diagnosed, or no preliminary hemostasis could be achieved in the ER. Hemodynamically stable patients with a negative history and clinical examination suspicious of vascular injury were admitted to the trauma surgical ward for observation. After 24 hours without complications the patient could be discharged home. All patients were instructed for alarm symptoms of vascular injury (loss of “vascular integrity” in the affected limb, e.g. expanding haematoma, loss of pulse, palor and coolness, or loss of sensation and function of the affected limb. Plus general signs of infection (erythema, swollen, warm)); if these occurred, they had to return to the hospital immediately.

Hemodynamically unstable patients were immediately transferred to the operating room. In actively bleeding patients hemorrhage control was attempted by using a tourniquet followed by Foley catheter balloon tamponade (FCBT). If hemorrhage control was not established, surgical exploration of the injured extremity had to follow immediately. If hemorrhage was controlled by FCBT, angiography or CTA was indicated after removal of an eventual tourniquet, in order to detect major arterial injury. If positive, patients should still be transferred to the operating room or treated by endovascular stenting or coiling. Without any arterial injury deemed in need of surgical or radiological interventional (RI) treatment, the patient should be observed for 24–48 hours, after which the Foley catheter was removed in the operating room. In case of re-bleeding, surgical intervention was performed.

## Results

A total of 668 patients (88.2% male; 33.8% GSWs) with PTE presented at the Emergency Department during the study period. After initial assessment, 512 patients were discharged home from the Emergency Department as the type and severity of their injury did not necessitate admission for observation or intervention. None of these patients returned to the hospital with late onset complications due to PTE.

Analysis of our prospective gathered trauma patient database revealed that a total of 156 patients were admitted after PTE. Stab wounds (SW) were found in 75 patients (10 women) and GSW in the remaining 81 patients (2 women). Characteristics of the patients and type and location of their sustained penetrating injuries are listed in Table 2. Sixteen patients underwent CTA as additional investigation to assess vascular integrity (Table 3). Although CTA should only be performed based on findings at physical examination with suspicion for vascular injury, according to the protocol, in four patients primary CTA was performed without relevant indication and without clinical signs of active bleeding. None of the four CTAs showed vascular injuries. Only one patient was initially treated with FCBT because of active bleeding. Subsequent diagnostic CTA showed minor arterial injury, which could be treated conservatively as no re-bleeding occurred after removal of the Foley catheter.

**Table 2:** Demographics of 156 patients admitted with penetrating extremity injury

Sex ratio (M:F)	144:12
Age, years (median; range)	27 (11–86)
**Penetrating extremity injury**	
Stab wound (female)	75 (10)
Gunshot wound (female)	81 (2)
**Extremity injury**	
*Vascular*	
Emergency exploration	14
Computed tomography angiography	8
*Fracture*	
X-ray^1^	14 (5)
*Neural*	
Physical examination^1^	22 (10)
**Concomitant penetrating injury**^**2**^	
Stab wound	45
Gunshot wound	22
Location	
Head	13
Neck	12
Chest	31
Abdomen	29
Thigh/Pelvis	3

1. Values in parentheses are numbers of surgical intervention because of injury;

2. Patients can have more than one concomitant penetrating injury.

**Table 3:** Indications for and results of vascular investigations

**Indication for investigation**	**CTA (n = 16)**
Absent or diminished pulses	1 (1)
Large hematoma	6 (5)
Foley catheter balloon catheter	1 (1)
Bruit	1 (1)
Proximity to major vessels	3 (0)
Not specified	4 (0)

Values in parentheses are numbers of additional investigations with positive findings on CTA, e.g. extravasation, stop, fistula.

CTA = Computed tomography angiography.

Twenty patients underwent emergency surgery because of ongoing bleeding or hemodynamic instability, not improving during initial resuscitation or because of extremity ischemia or specific findings at CTA. Another 20 patients underwent surgery for reasons mentioned in Table 4. Overall, 22 (14%) patients that were admitted underwent exploration of the extremity for vascular injury. In 12 of these patients reconstruction of vascular injury with use of a venous graft was performed, instead of primary repair or suture ligation. No patients were treated primarily by radiological intervention. Six patients underwent surgery to repair traumatic fractures and another nine patients underwent surgery because of nerve injury. In one patient the plastic surgeon joined the trauma surgeon during fracture care surgery to repair neural injury (Table 4). Primary fasciotomy was performed in four patients: one underwent fasciotomy to treat an acute compartment syndrome, the others underwent pre-emptive fasciotomy after vascular reconstructive surgery (n = 2) and nerve injury repair. Fractures of the extremities after penetrating injury were almost exclusively found after GSW (n = 13). One metacarpal fracture was found in a patient with SW.

**Table 4:** Indications for surgical intervention

**Indication for emergency exploration**	**20**
Active hemorrhage or shock	9
Absent pulses	5
Vascular injury found at CTA	6
**Indication for early surgery**	**20**
Vascular injury found at CTA	2
Fracture	5
Neural injury	9^1^
Wound management	2
Removal of bullet	1
Fasciotomy of the lower leg	1

1. One patient who underwent exploration because of nerve injury also was operated on to repair a metacarpal fracture.

CTA = Computed tomography angiography.

In 134 patients conservative observational strategy for vascular symptoms could be initialized after PTE. This equals 86% of admitted patients and 97% of all patients presented at the Emergency Department after PTE. After conservative observation, two (1.5%, or 0.3%, respectively) of these patients subsequently needed an intervention to treat (late onset) vascular complications (Table 5). In one patient emergent repair of the deep femoral artery was complicated by the formation of an arterio-venous fistula discovered after clinical observation and additional CTA, which was treated by endovascular coiling. The other patient returned with a false aneurysm of the popliteal artery several months later, which was missed at CTA during first admission. This patient was successfully operated on by the vascular surgeon.

**Table 5:** (Long-term) complications that were initially missed or had severe consequences

**Initial treatment**	**Complication**	**Consequence/result**
Stab wound		
*Exploration*	-Brain-injury due to exsanguination (n = 2)	Death
	-Femoral nerve injury	Weakness leg
	-Arterio-venous fistula after femoral a. repair	Coiling
*Conservative*	-Brachial plexus lesion	Limp/ weakness arm
	-Median nerve lesion	Ape hand deformity
	-Ulnar nerve injury (n = 2)	Paraesthesia and weakness
Gunshot wound		
*Exploration*	-Leg length difference after femur fracture	Surgical correction
	-Sciatic nerve injury after femoral a. repair	Leg pain and foot weakness
	-Hip joint disarticulation after femoral a. injury and femur fracture	Wheelchair bound
	-Peroneal nerve injury after compartment syndrome	Foot drop
	compartment syndrome after popliteal a. repair (n = 2)	
*Conservative*	-False aneurysm popliteal a.	Surgical repair
	-Erysipelas foot due to bullet	Surgical exploration
	-Ulnar nerve injury	Claw hand

Two patients (both SW) died of diffuse axonal injury and post anoxic encephalopathy after exsanguination due to penetrating chest and extremity injury. Besides, the complications mentioned above, long-term extremity related complications were loss of function or other deformity (n = 9) including two patients with peroneal nerve injury caused by delayed compartment syndrome treatment, late onset infection and severe wound healing problems resulting in hip exarticulation (n = 1; combined injury of femoral artery and proximal femur).

## Discussion

In the Netherlands, as in the rest of Western Europe, the incidence of penetrating injury is rather low. Due to the low incidence it is not possible for a trauma surgeon to get extensive experience with the management and treatment of this kind of trauma, causing obscurity, disagreement in diagnostic and treatment options, and an insufficient or incomplete management of this trauma patient. All together, inexperience in assessment of patients with PTE might increase the risk of mistakes and may hamper outcome.

In trauma centers that treat a higher number of patients with penetrating trauma, SNOM is becoming more and more accepted. SNOM is based on clinical examination and additional investigations (on indication). Together they have shown to be a reliable indicator of clinically significant injury, with a sensitivity and specificity of 99% and a negative predictive value of 99% [6,10]. The management protocol for assessing and treating patients with PTE is based essentially on hemodynamic status, together with a thorough physical examination. Adjuvant CTA is only indicated based on hard and subtle signs of vascular injury found during clinical assessment in hemodynamically stabilized patients. CTA is a reliable and accurate investigation with a sensitivity and specificity of 95% and 100% respectively, a positive predictive value of 100% and a negative predictive value of 98% [11-13]. Therefore CTA is more and more becoming the diagnostic tool of choice during initial evaluation of stable patients with suspected vascular injury, including patients after PTE [13,14]. The combination of FCBT and CTA could also diminish the rate of negative explorations and iatrogenic injuries. In one patient an actively bleeding groin was successfully controlled by FCBT. Subsequent CTA revealed no indication for surgical exploration, and after two days the catheter was removed without rebleeding.

In the present study the SNOM protocol for penetrating extremity injury was correctly executed with good persistence. Only four out of 124 admitted patients with no signs of vascular injury still underwent CTA. None showed signs of vascular lesions, and all four were successfully treated conservatively. Vascular observational management after PTE was applied in 86% of admitted patients without (n = 126) or after CTA (n = 8) assessment. During follow up only one (0.7%) of the patients who were conservatively treated and observed returned with symptoms of a false aneurysm several months later. This indicates that initial conservative management (or SNOM) of patients with PTE is feasible and safe.

Although the majority of patients presented at the Emergency Department with supposed PTE are not seriously injured and can be discharged after physical examination and treatment of wounds, up to a quarter of patients should be admitted for observation, additional investigations or surgical treatment. The total surgical treatment rate of the latter group was 24% (22 vascular injuries, five fractures, 10 exclusively neural injuries), indicating that PTE should be considered a serious trauma which requires intensive and thorough assessment of the extremities. PTE is frequently accompanied by other penetrating injuries (in this study in 43% of cases), that possibly needs to be managed first or distracts the physician’s attention away from the injuries of the extremities. Eventually missed or even delayed assessment of PTE may significantly impair outcome of the patient [15,16].

In the present study, seven patients (5%) who were treated conservatively showed symptoms of nerve injury that were missed during the initial hospital stay. Although the larger part of nerve injuries cannot be treated, it is important to recognize these injuries at initial assessment, in order to adequately inform patients and provide supportive treatment. These are important factors in the rehabilitation process after penetrating trauma, especially for patients with prolonged or definitive impairment of the extremity [17].

Not only is it important to recognize nerve injury at initial assessment, it is of vital importance to prevent nerve injury in a later stage of treatment. Of all 12 patients that underwent primary vascular repair, only two underwent fasciotomy during the same vascular-reconstructive operation in order to prevent compartment syndrome. In two (20%) patients who had not undergone fasciotomy, compartment syndrome after revascularisation of the leg was diagnosed too late, resulting in persistent peroneal nerve injury. In other words, a patient sustaining PTE should not only be intensively reassessed several times during conservative treatment, but also after surgical treatment, not only for vascular injury, but nerve injury as well. Besides, pre-emptive fasciotomy is advised, in patients sustaining a combination of arterial and venous injury, multiple or complex fractures and an ischemia time longer than six hours [18,19], as continuous compartment pressure-monitoring is not reliable. Blood flow should be restored as soon as possible by using a shunt. After initial shunting, fractures should be rigidly stabilized using external fixation devices, in order to perform definitive vascular repair with a tension free (venous) interposition graft [20]. Since these repairs usually take a fair amount of time, there is a serious threat of compartment syndrome after revascularisation. Therefore, a pre-emptive fasciotomy is highly recommended.

In summary, the low failure rate in this study validates the SNOM protocol for initial management of PTE. Clinical examination of the injured extremity is a reliable diagnostic approach for excluding vascular injury. It is important to assess for possible nerve injuries, both pre- and post operatively, as these injuries are frequently missed and might result in long-term disability.

## Competing interests

The authors declare that they have no competing interests.

## Authors’ contribution

OW: Writing of the article, data interpretation, approved final version, EML: Study design, data interpretation, critical revision, approved final version, WH: Data collection, approved final version, JAH: Data collection, literature search, approved final version, JV: Study design, data analysis, data interpretation, writing, approved final version.
